# Distribution of epiphyseal nutrient foramina in the distal femur: Implications for anterior knee joint denervation

**DOI:** 10.1016/j.inpm.2025.100708

**Published:** 2025-11-25

**Authors:** John Tran, Alicia J. Chung, Ian bell, Brent Lanting, Zachary L. McCormick, Eldon Loh

**Affiliations:** a Division of Anatomy, Department of Surgery, University of Toronto, Toronto, Ontario, M5S 1A8, Canada; b Department of Physical Medicine and Rehabilitation, Parkwood Institute, London, Ontario, N6C 0A7, Canada; c Department of Orthopedic Surgery, University Hospital, Western University, London, N6A 5A5, Canada; d Department of Physical Medicine and Rehabilitation, University of Utah School of Medicine, Salt Lake City, 84108, United States

**Keywords:** Anatomy, Osteology, Knee denervation, Genicular, Diagnostic block

## Abstract

**Background:**

Sensory afferents supplying subchondral bone could mediate pain from the knee joint. Intrinsic innervation originates externally and follows blood vessels through nutrient foramina. Therefore, targeting the intrinsic innervation of subchondral bone can be achieved by capturing extrinsic innervation prior to their entry into the nutrient foramina. Understanding of extrinsic innervation of the knee joint as well as the distribution of the epiphyseal nutrient foramina are important. Currently, the distribution of nutrient foramina has not been analyzed. The objective of this osteological study was to quantify the distribution of nutrient foramina in the distal femur to inform knee joint denervation strategies.

**Methods:**

A convenience sample of 19 bony femurs was used in this study. The distal end of each specimen was photographed to obtain standardized lateral, medial, and anterior views. The location of nutrient foramina was documented. Each photograph was imported into ImageJ and the distribution of nutrient foramina was quantified.

**Results:**

Location of epiphyseal nutrient foramina was variable on distal femur. Laterally, distribution of nutrient foramina showed percentages of 11.5 %, 44.7 %, 36.5 %, and 7.3 % in the first, second, third, and fourth quadrants, respectively. Distribution on the medial distal femur showed percentages of 12.4 %, 40.4 %, 35.5 %, and 11.5 % in the first, second, third, and fourth quadrants, respectively. Anteriorly, distribution showed a difference between the medial and lateral halves with percentages of 71.1 % and 28.9 %, respectively.

**Conclusions:**

Epiphyseal nutrient foramina are important conduits that enable extrinsic innervation to enter and supply the subchondral bone. The location and distribution of the nutrient foramina of the distal femur reported in this study can be used to optimize nerve blocks and denervation techniques to manage chronic knee joint pain from osteoarthritis.

## 1 Introduction

Knee joint denervation is a common procedure to treat chronic knee pain related to osteoarthritis (OA) [1]. However, clinical results remain to be further optimized. Recent anatomy studies have expanded the understanding of knee joint innervation [[2], [3], [4]]. This new knowledge has led to the development of novel denervation techniques [5,6], including a radiofrequency ablation (RFA) protocol with up to 10 targets [7,8], to enhance nerve capture rate and potentially improve pain relief outcomes. However, the most feasible approach and optimal number of targets remains to be further investigated.

More recently, a new concept of intrinsic innervation of the epiphyseal ends of the distal femur and proximal tibia has emerged [9], and along with it, a call for specific research addressing it [10]. Conceivably, the presence of sensory afferents supplying subchondral bone (intrinsic innervation) could mediate transmission of pain sensation from the knee joint. Therefore, the origin and course of these intrinsic innervations may play an important role in determining optimal knee denervation approaches.

**Fig. 1:**
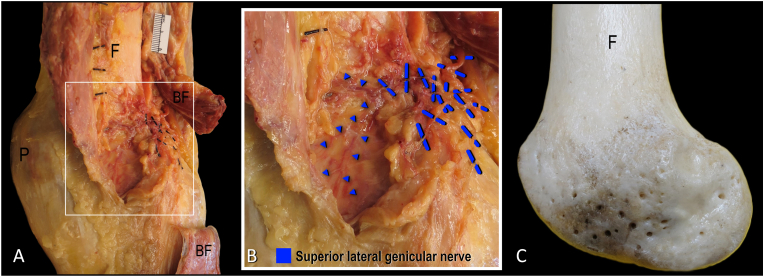
**Course of nerves prior to entering nutrient foramina to supply subchondral bone. A.** Articular branches supplying lateral knee. **B.** Articular branches of superior lateral genicular nerve coursing along periosteum of lateral aspect of femoral condyle towards nutrient foramina (blue arrowheads). **C.** Location of epiphyseal nutrient foramina that provide channel for blood vessels and nerves to access and supply subchondral bone. BF indicates biceps femoris; F, femoral shaft; P, patella. (For interpretation of the references to color in this figure legend, the reader is referred to the Web version of this article.)

In previous anatomical literature, it has been reported that the intrinsic innervation of human bones originates externally and follows the course of blood vessels through nutrient foramina [11,12]. The origin and course of these intrinsic sensory afferents have been previously described, where the “… fibers entering the bones are derived from nerves traveling in the periosteal plexus …” and “… plexuses in the joint capsule extend to the underlying bone and enter the cortex through foramina” (Fig. 1) [11]. It has also been reported that “no nerve fibers permeated the epiphysis to diaphysis” [12]. This suggests that the innervation of the subchondral bone originates from extrinsic innervation that enters into the bone through epiphyseal nutrient foramina often accompanying vascular structures (Fig. 1C). Therefore, targeting the intrinsic innervation of subchondral bone can be achieved by capturing extrinsic innervation prior to their entry into the nutrient foramina. Understanding the anatomy of the periosteal and capsular plexuses supplying the knee joint [2,3] as well as the distribution of the epiphyseal nutrient foramina are important considerations for knee denervation. Currently, the distribution of epiphyseal nutrient foramina has not been analyzed, which limits optimization of denervation techniques to better capture nerves supplying intrinsic innervation to the subchondral bone. Therefore, the objective of this osteological study was to quantify the distribution of nutrient foramina in the distal femur to inform knee joint denervation strategies.

## 2 Material and methods

This osteological study was conducted in the Division of Anatomy at the University of Toronto. Approval was received from the University of Toronto Health Sciences Research Ethics Board (#27210).

### 2.1 Osteology specimens and photography

Bony femurs were procured from the anatomical collection in the Division of Anatomy at the University of Toronto. Specimens with damage to the distal femur were excluded. A convenience sample of 19 specimens was included in this study. The distal end of each femur was photographed to obtain standardized lateral, medial, and anterior views. The photographs were subsequently analyzed to determine the distribution of epiphyseal nutrient foramina for each specimen.

### 2.2 ImageJ quantification and data analysis

Each photograph was imported into ImageJ, an open-source image processing software. Using built-in tools available in ImageJ, each photograph was converted into an 8-bit format to enable further processing and analysis. Using the 8-bit converted image, the color threshold function was used to label/segment the nutrient foramina on the lateral, medial and anterior images of the distal femur. Color threshold-based segmentation of nutrient foramina was possible because the canal, penetrating the bone, generates a shadow that has a distinct color intensity from the cortex (Fig. 1C).

**Fig. 2:**
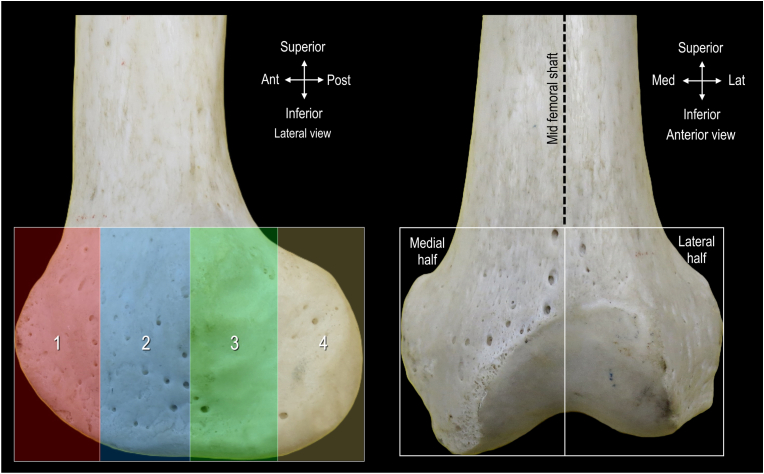
**Methodology to quantify distribution of nutrient foramina on distal femur.** 1 indicates first quarter; 2, second quarter; 3, third quarter; 4, fourth quarter.

To facilitate description of the distribution of nutrient foramina, a rectangular region of interest was defined on the distal femur. On the lateral and medial views, the width of the distal femur at its widest point, in the anterior to posterior direction, was used to determine the horizontal boundaries of the rectangular region of interest. Vertical boundaries spanned the junction of the shaft of the femur and femoral condyle to the most inferior part of the condyle. This rectangular region of interest was further divided into 4 equal quadrants for quantification and comparison (Fig. 2).

On the anterior view, the horizontal boundaries of the rectangular region of interest were defined by the length between the medial and lateral epicondyles. The vertical boundaries spanned the junction of the shaft of the femur and femoral condyle to the most inferior part of the condyle. The region of interest in the anterior view was divided into equal medial and lateral halves using a line drawn along the length of the femoral shaft (Fig. 2).

Using the ImageJ box selection tool, the rectangular region of interest was selected on each image, and the total number of nutrient foramina was quantified. Next, on lateral and medial images, each quadrant was individually selected with the box selection tool and nutrient foramina within the region were quantified. This was completed for all 19 specimens and a percentage of the total nutrient foramina in each quadrant was determined. Similarly, in the anterior images, the percentage of total nutrient foramina in the anterior aspect of the distal femur was quantified for the medial and lateral halves. The location of the nutrient foramina was described, and the distribution percentages were reported.

## 3 Results

A total of 57 images were taken and analyzed from 19 bony femurs. There was variability in the location and number of nutrient foramina within the region of interest in the distal femur across different specimens.

**Fig. 3:**
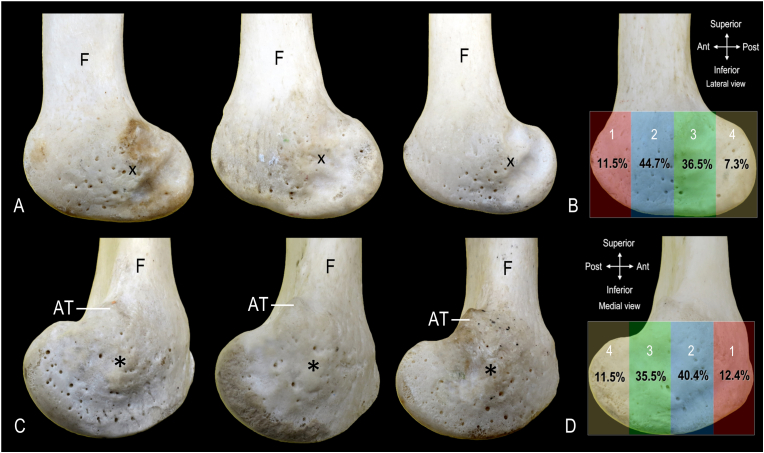
**Distribution of nutrient foramina on lateral and medial aspects of distal femur. A.** Lateral view of distal femur showing location of nutrient foramina in 3 different specimens. **B.** Distribution map of nutrient foramina on lateral aspect of femoral condyle calculated from 19 specimens. **C.** Distribution of nutrient foramina on distal femur, medial view. **D.** Distribution map of nutrient foramina on medial femoral condyle calculated from 19 specimens. AT indicates adductor tubercle; F, femoral shaft; *, medial epicondyle; X, lateral epicondyle; Red box, first (1) quarter; Blue box, second (2) quarter; Green box, third (3) quarter; Yellow box, fourth (4) quarter. (For interpretation of the references to color in this figure legend, the reader is referred to the Web version of this article.)

On the lateral aspect of the distal femur, the locations of epiphyseal nutrient foramina were variable in their vertical and horizontal positions (Fig. 3A). The majority of the epiphyseal nutrient foramina were found to be anterior to the lateral femoral epicondyle. Quantification of the distribution of nutrient foramina based on quadrants showed percentages of 11.5 %, 44.7 %, 36.5 %, and 7.3 % in the first, second, third, and fourth quadrants, respectively (Fig. 3B). Similarly, on the medial aspect of the distal femur, there was variability in the location of the nutrient foramina (Fig. 3C). The distribution of the foramina based on quadrants demonstrated percentages of 12.4 %, 40.4 %, 35.5 %, and 11.5 % in the first, second, third, and fourth quadrants, respectively (Fig. 3D).

**Fig. 4:**
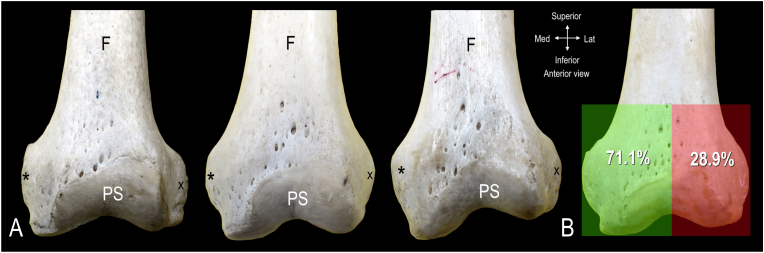
**Distribution of nutrient foramina on anterior aspect of distal femur. A.** Nutrient foramina on 3 different left femurs. **B.** Distribution map of nutrient foramina on anterior aspect of distal femur quantified from 19 specimens. F indicates femoral shaft; PS, patella surface; *, medial epicondyle; Green box, medial half; Red box, lateral half. (For interpretation of the references to color in this figure legend, the reader is referred to the Web version of this article.)

In the anterior view, the locations of the nutrient foramina within the region of interest were variable but consistently found superior to the patellar surface of the femur (Fig. 4A). Quantification of the distribution of the nutrient foramina showed a difference between the medial and lateral halves with percentages of 71.1 % and 28.9 %, respectively (Fig. 4B).

## 4 Discussion

**Fig. 5:**
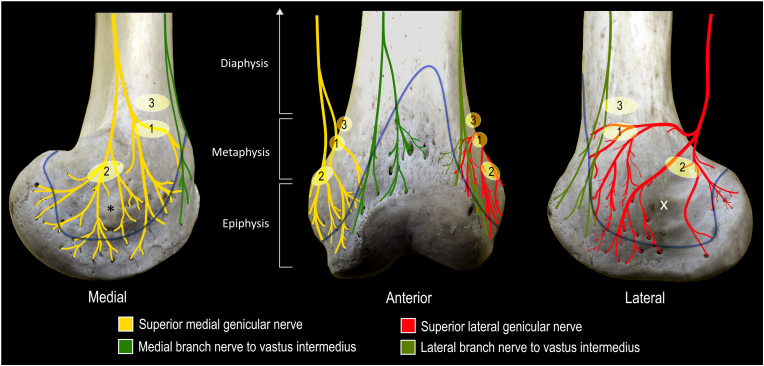
**Schematic illustration of one possible extrinsic innervation pattern supplying intrinsic innervation to subchondral bone by coursing through nutrient foramina.** 1, classical target site for superior medial and lateral genicular nerves, 2, previously published supplementary target for superior medial and lateral genicular nerves [24]; 3, previously published secondary landmark for bipolar lesion [21]; blue curve, approximate attachment of joint capsule; X, lateral epicondyle; *, medial epicondyle. (For interpretation of the references to color in this figure legend, the reader is referred to the Web version of this article.)

The intrinsic innervation of bone, in the context of joint pain, has emerged as an intriguing consideration for pain interventionalists [9]. Previous anatomical literature has provided histological evidence supporting the important role of nutrient foramina as conduits for extrinsic innervation to enter and supply intrinsic innervation to the subchrondal bone (Fig. 5) [11,12]. In the current osteological study, the location and distribution of the epiphyseal nutrient foramina were described and quantified. The relevance of the study findings to previous anatomical literature, diagnostic blocks, and knee denervation approaches are discussed below.

### 4.1 Relevance to previous anatomical literature

In a previous anatomical study, the innervation of the medial and lateral knee joint was investigated [3]. The anatomical dissections were completed to a very detailed level, identifying and describing where nerves ramified along the joint capsule and periosteum of the distal femur. Importantly, the periosteal nerve fibers were found to course with blood vessels (Fig. 1B) [3]. This was consistent with previous reports, which described nerves and their accompanying vessels passing through nutrient foramina to enter the cortex of bone [11]. In the current study, the location and distribution of the epiphyseal nutrient foramina on the distal femur were investigated and described. This anatomical knowledge is important as the nutrient foramina represent termination points for the previously described extrinsic innervation to this area and allows for a description of the position of nerves just prior to their entry into the cortex of the femoral epiphysis (Fig. 5).

In a review article published by Devor, the importance of nutrient foramina was highlighted, and intrinsic innervation to the subchondral bone was depicted in schematic diagrams [9]. The diagrams included nerve fibers coursing from the diaphysis into the epiphysis of the femur. Although this was likely a basic visual to highlight the importance of intrinsic innervation, it should be noted that previous anatomical evidence has not described nerve fibers that pass from the diaphysis to the epiphysis [12]. Anatomically, previous studies suggest that subchondral bone innervation derives from extrinsic innervation entering through the epiphyseal nutrient foramina, and not from nerves fibers traversing internally from the diaphysis proximally into the epiphysis distally.

Consequently, interventions directly targeting the subchondral bone of the distal femur (i.e. intraosseous injections/ablation) are likely unnecessary. Instead, targeting the intrinsic innervation could be accomplished by capturing its upstream extrinsic innervation prior to entry of the extrinsic nerves into the foramina. By considering the location and distribution of epiphyseal nutrient foramina in this study with the previously described location of nerves around the knee joint [[2], [3], [4]], procedures for capturing the extrinsic innervation, and the intrinsic innervation as a consequence, could be optimized without violating the joint capsule. This represents a less invasive approach to target the intrinsic innervation of the subchondral bone and reduce risk of septic arthritis [13].

### 4.2 Implications for patient selection using diagnostic blocks and chemical neurolysis

Genicular diagnostic nerve blocks are commonly used to select patients for knee joint denervation [[14], [15], [16]]. There have been reported differences in the predictive efficacy of genicular nerve block on knee denervation treatment success. Previous studies by Chen et al. [14] and Kose et al. [15] found a positive relationship between response to nerve block and better RFA treatment outcomes. It has also been reported that there is no difference in RFA treatment success with or without utilization of prognostic nerve block [16,17]. A potential explanation for this is due to differences in procedural techniques. In a recent practice survey on knee RFA, it was concluded that “significant variability exists in prognostic block protocols” [18].

Based on the findings of the current study, the 2nd and 3rd quadrants on both lateral and medial aspects of the distal femur had the highest distribution percentages of nutrient foramina. In these quadrants, the concentration of extrinsic nerve branches will be higher, as they are just about to enter subchondral bone through the nutrient foramina. Therefore, depositing and concentrating local anesthetic in these two quadrants may be more appropriate to optimize patient selection and to minimize false negatives from genicular nerve block. On the anterior aspect of the femur, the distribution percentage of the nutrient foramina was 71.1 % and 28.9 % on the medial and lateral halves, respectively. This finding suggests that injection of local anesthetics should cover the area above the patellar surface with greater concentration on the medial half, which may lead to better selection of patients for knee denervation.

The results of the current study, combined with anatomical knowledge of the course of articular branches, can also be used to optimize target sites for injection of neurolytic injectables (e.g. phenol), ensuring adequate coverage of extrinsic innervation without violation of the joint capsule. Based on assessment of previously published fluoroscopic images with contrast enhanced injections, injectate was concentrated superior to the current study's region of interest [19,20]. Although this may capture some articular branches that likely terminate in anteriorly located nutrient foramina at a more proximal location (i.e. lateral and medial branches of nerve to vastus intermedius), extrinsic innervation located at the level of the femoral condyles (i.e., most branches of the superior medial and lateral genicular nerves) would be spared. Based on the findings of the current study, if contrast is used with injection, optimal localization of the neurolytic agent is within the 2nd and 3rd quadrants on the lateral and medial aspects of the distal femur/femoral condyle.

### 4.3 Implications for RFA protocols

Due to the extensive nerve supply to the knee joint, interest in expanding the number of RFA lesion sites has emerged. There have been several descriptions of clinical protocols using bipolar lesions [21] and multi-target techniques ranging from 6 RFA lesions [5] to 10 [7,8,22,23]. The optimal RFA lesion approach remains to be further investigated clinically. Based on the findings of the current study, however, lesion placements should prioritize targeting nerves that supply the 2nd and 3rd quadrants on the lateral and medial aspects of the distal femur to maximize denervation of the subchondral bone.

In a recent case report/teaching image, RFA targets were adjusted based on observed contrast flow during genicular nerve blocks. Contrast was found to flow inferior and posterior over the femoral condyle after injection at the classical genicular target site [24]. When RFA was performed, supplementary targets were therefore added posterosuperior to the lateral and medial femoral epicondyles, in addition to classical target sites [24]. The addition of supplementary lesions to the classical superomedial and superolateral target sites was previously suggested in a 2020 study [3]. Lesion placements in this case report, and as suggested in the 2020 anatomical study, will likely capture more of the extrinsic innervation that enters the nutrient foramina in the 2nd and 3rd quadrants (Fig. 5, Lesion 1 & 2). This suggests that RF lesion placement in these areas may be more optimal. However future study to assess safety and possible adverse events (i.e., avascular necrosis) is warrant.

In contrast, a previous study on knee RFA utilizing a bipolar technique with placement superior to the femoral condyles reported no significant difference in pain relief at 12 months [21]. Lack of success may be due to placement that would not capture the extrinsic innervation supplying the 2nd and 3rd quadrants, where nutrient foramina were found to be concentrated in this study (Fig. 5; Lesion 3). Thus, an understanding of the distribution of nutrient foramina and the extrinsic innervation of the knee are important in optimizing RFA lesion placement. Further clinical investigation is required to assess the effectiveness of different RFA lesioning techniques, particularly in the context of the new anatomical knowledge regarding the locations of nutrient foramina.

### 4.4 Limitations

The current study is limited to a small sample size and does not encompass all potential population variability and degenerative changes that may impact the distribution of nutrient foramina. However, the current study's sample size is greater than the number that was previously recommended [25]. Another limitation is that the bones within the anatomical collection in the Division of Anatomy at the University of Toronto have been de-identified and therefore analysis based on sex, age, and ethnicity is not possible. As an osteological study, all clinical implications discussed require further investigation.

## 5 Conclusions

Epiphyseal nutrient foramina are important conduits that enable extrinsic innervation and vasculature to enter and supply the subchondral bone. In the current study, the location and distribution of the nutrient foramina of the distal femur have been described and quantified. These anatomical findings can be used to optimize nerve blocks and denervation techniques to manage chronic knee joint pain.

## Disclosures

John Tran, PhD has research grants from Avanos Medical and FUSMobile (paid directly to Lawson Research Institute), and also consultancies with Brixton Biosciences, and Merz Therapeutics.

Zachary L. McCormick, MD serves on the Board of Directors of the International Pain and Spine Intervention Society (IPSIS), has research grants from Avanos Medical, Boston Scientific, Relievant Medsystems, Saol Therapeutics, Spine Biopharma, SPR Therapeutics, Stratus Medical (paid directly to the University of Utah), and also consultancies with Avanos Medical, Saol Therapeutics, Stryker, and OrthoSon (relationships ended).

Eldon Loh, MD has research grants from Avanos Medical and FUSMobile (paid directly to Lawson Research Institute), and also is a consultant with Brixton Biosciences.

## Funding

This study did not receive funding from agencies in the public, commercial, or not-for-profit sectors.

## Declaration of competing interest

The authors declare the following financial interests/personal relationships which may be considered as potential competing interests: John Tran reports a relationship with Brixton Biosciences Inc that includes: consulting or advisory. John Tran reports a relationship with Merz Therapeutics GmbH that includes: consulting or advisory. Eldon Loh reports a relationship with Brixton Biosciences Inc that includes: consulting or advisory. Zachary L McCormick reports a relationship with Avanos Medical Inc that includes: consulting or advisory. Zachary L McCormick reports a relationship with Saol Therapeutics that includes: consulting or advisory. Zachary L McCormick reports a relationship with Stryker Corporation that includes: consulting or advisory. Zachary L McCormick reports a relationship with OrthoSon that includes: consulting or advisory. If there are other authors, they declare that they have no known competing financial interests or personal relationships that could have appeared to influence the work reported in this paper.
